# Novel Second-Order Fully Differential All-Pass Filter Using CNTFETs

**DOI:** 10.3390/mi14101873

**Published:** 2023-09-29

**Authors:** Muhammad I. Masud, Iqbal A. Khan, Syed Abdul Moiz, Waheed A. Younis

**Affiliations:** 1 Department of Electrical Engineering, College of Engineering and Islamic Architecture, Umm Al-Qura University, Makkah 21955, Saudi Arabia; iqbalakhan19@gmail.com (I.A.K.); wamuhammad@uqu.edu.sa (W.A.Y.); 2 Device Simulation Laboratory, Department of Electrical Engineering, College of Engineering and Islamic Architecture, Umm Al-Qura University, Makkah 21955, Saudi Arabia; sasyed@uqu.edu.sa

**Keywords:** APF, phase angle, chirality, CNTFET, fully differential

## Abstract

In this paper, a new carbon nanotube field effect transistor (CNTFET)-based second-order fully differential all-pass filter circuit is presented. The realized filter uses CNTFET-based transconductors and grounded capacitors. An active-only second-order fully differential all-pass filter circuit topology is also presented by replacing the grounded capacitance with a CNTFET-based varactor to achieve filter tunability. By controlling the varactor capacitance, active-only second-order fully differential all-pass filter tunability in the range of 15 GHz to 27.5 GHz is achieved. The proposed active-only circuit works on -oltage, low-power dissipation and high tunable pole frequency. The realized circuit operations are verified through the HPSPICE simulation tool. Deng’s CNTFET model is utilized to verify the filter performances at the 16 nm technology node. It is seen that the proposed filter simulation justifies the theoretical predictions and works efficiently in the deep-submicron technology.

## 1. Introduction

Fully differential topologies enjoy several advantages over single-ended circuits, such as increased immunity to external noise, suppression of power supply noise, lower harmonic distortion and larger dynamic range [1]. Due to the trend towards low-voltage and low-power mixed-mode signal circuits, there is a growing interest in designing fully differential circuit topologies. Mixed-mode signal circuits simplify the design, enable compactness and reduce cost; however, signal interference from digital-blocks to analog-blocks -remains a challenging problem [2]. For these applications, fully differential circuits are recognized as a better solution, as these provide immunity to digital noise [3]. Subsequently, several voltage mode and current mode fully differential circuits have been reported in the literature [1,2,3,4,5,6,7,8,9,10,11].

In analog signal processing circuits, one of the most discussed function blocks is the all-pass filter (APF). APFs, also called phase shifters, are widely utilized for phase equalization while keeping the gain constant over the desired frequency range. APF finds applications in the realization of several high-Q frequency selective circuits, oscillators and radio frequency beamformers [12]. In the open literature, various voltage mode [13,14,15,16,17,18,19,20] and current mode [21,22] fully differential first-order APFs are reported. However, very limited circuit topologies are reported for second-order fully differential APFs in the technical literature [2,23,24]. The circuit discussed in [2] utilizes two negative second-generation current conveyors as an active building block (ABB), along with three floating resistors and three floating capacitors for the realization of a second-order fully differential APF. Another second-order fully differential APF topology, based on single differential voltage current conveyor, three floating resistors and three capacitors, with one bring grounded, has been reported in [23]. In [24], a second-order fully differential APF circuit with two grounded resistors, two grounded capacitors and three ABBs has been demonstrated. The circuit employed two differential difference current conveyors and one second-generation current conveyor as ABBs. Most of these APFs use large active and passive component counts with complex matching constraints and limited frequency range of operation.

It is to be noted that second-order fully differential APF topologies realized in the open literature [2,23,24] are based on bulk CMOS technology, which faces numerous challenges due to incessant focus on transistor scaling in the nanometer regime to further validate the Moore’s law. These challenges include short channel effects, high field effects, boron penetrations, lithographic limitations, polysilicon depletion, gate leakage, enlarged heat production, etc. [25,26]. There is a critical need to replace conventional bulk semiconducting circuit technologies with other robust and reliable technologies to efficiently work in the nanometer regime. Recently, numerous devices have been introduced, like CNTFET, strained-Si FET, double-gate FET and FinFETs [27]. Among these, CNTFET is considered as a promising device as it offers near ballistic transport of carriers, excellent electrostatic control, lesser parasitics, low power dissipation, larger thermal conductivity, larger drive current and higher cutoff frequency, to name a few [25,26,27].

Since CNTFET’s introduction as a possible alternative, very limited work has been done in the analog filtering domain [12,19,20,25,26,28,29,30]. Voltage-mode first-order active-only APF based on a single inverting voltage buffer (IVB) and CNTFET-based varactor has been reported in [12]. The tunability of pole frequency was achieved through variation of varactor capacitance. Similarly, in [25], another voltage-mode first-order APF based on a single CNTFET-based IVB, one capacitor and one voltage controlled resistor has been presented. Although both circuits [12,25] consume considerably low power and achieve high tunable pole frequency, these APFs are suitable for single-ended operation modes. CNTFET-based circuit solutions for first-order voltage-mode multifunctional filters [30], second-order voltage-mode multifunctional filters [28,29] and third-order high-pass butterworth filters [26] have also been reported, but the majority of these circuits are not suitable for fully differential applications. Some CNTFET-based voltage-mode first-order APFs suitable for fully differential applications have been presented in [19,20]. The circuit reported in [19] utilized three floating resistors, three floating capacitors and two CNTFET-based digitally controlled differential voltage current conveyors. Although the circuits achieve a reconfigurable pole frequency control, the reported topology uses excessive numbers of active and passive components. It is to be noted that the majority of CNTFET-based filters are suitable for single-ended applications or first-order fully differential responses; however, no circuit solution for second-order fully differential APFs is available in the open literature.

This paper aims to realize new fully differential APF topologies with compact circuit configurations for low-voltage, low-power and high-frequency analog signal processing applications. The realized circuit employs three positive transconductors, five negative transconductors, and two grounded capacitors. An active-only second-order fully differential APF is also derived from the first proposed APF by replacing the grounded passive capacitors with CNTFET-based varactors. The proposed APFs are designed and simulated in HSPICE by utilizing Deng’s CNTFET model. The realized active-only second-order fully differential APF successfully demonstrates a wide tunable pole-frequency range of 15 GHz to 27.5 GHz, which is considerably large comparatively to available circuits of fully differential second-order APFs. The realized topologies work on a low supply voltage of 0.7 V. The simulations of proposed topologies successfully verify the theory. This paper contains seven sections: An overview of CNTFET is given in Section 2. Section 3 demonstrates the realized novel APFs. The non-ideal analysis is conducted in Section 4. Section 5 demonstrates the circuit design and verification. Section 6 shows the comparison of the work with other relevant second-order fully differential APFs. The overall conclusion on the subject is given in Section 7.

## 2. Carbon Nanotube Field Effect Transistors

Carbon nanotube (CNT) is one of the most amazing materials, which has diverse applications and covers nearly all fields of semiconductor-based electronic devices [31,32,33]. A CNT is nothing but a graphene-based sheet in the form of a cylindrical pipe that has a diameter typically in the range of nanometers for most cases. However, it is much harder than steel and offers many unique electrical, optical, chemical, biomedical and mechanical properties, both in pure form and in composite materials, which really make them ideal for many electronic applications [34,35,36].

**Figure 1:**
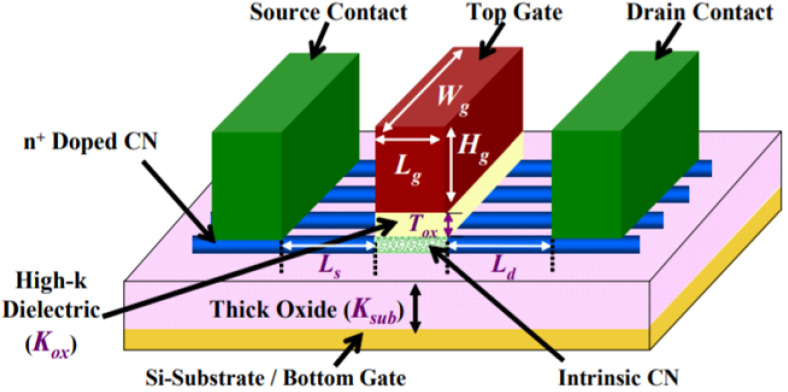
CNTFET three-dimensional view.

CNTs are inherently classified as either single-walled carbon nanotubes (SWCNTs) or multi-walled carbon nanotubes (MWCNTs). The SWCNTs are a special case of carbon-based sp^2^ hybridized very similar to the fullerene. The SWCNT-based field effect transistor CNTFET is an emerging electronic device that has the much potential to fulfill Moore’s requirements of scaling for the next-generation electronics industry. From a device structure point of view, CNTFET is very similar to the MOSFET, where either a single CNT or array of CNTs are used as a channel to replace the bulk region of the conventional MOSFET. The CNTFET-based structures enjoy low voltage and low power consumption, and can be easily scaled down and integrated with current Si-based CMOS technology [37,38,39,40]. Figure 1 shows the schematics of a CNTFET [25].

The diameter (*D_CNT_*) of SWCNTs in the channel can affect the overall threshold voltage (*V_th_*), which is a very crucial parameter to control the behavior of CNTFET. It is directly related to the chirality vectors (*n*_1_, *n*_2_) of SWCNT and can be expressed as [41,42]:

(1) $$D_{CNT}=\frac{a\sqrt{\left({n_{1}}^{2}+{n_{2}}^{2}+n_{1}n_{2}\right)}}{\pi }$$

(2) $$V_{th}=\frac{aV_{\pi }}{\sqrt{3}qD_{CNT}}$$

where *a* = 2.49 Å is the lattice constant, *V_π_* = 3.033 eV is the bond energy and *q* is the charge of the electron.

Due to the complex nature of CNTFET, creating an efficient, accurate and compact model predicting the experimental current-voltage responses is really a challenging task. Over the past few decades, a great deal of research has been done on the device modeling and simulation of CNTFETs. Despite these significant efforts, there is presently no full-device all-purpose compact model for CNFETs documented in the literature, mostly due to the lack of complete knowledge of the charge transport mechanism of these devices. On the other side, a computationally efficient and accurate compact model explaining the CNTFET behavior is crucial in the design of both analog and digital circuit applications for diverse electronic and communication system applications. Various CNTFET models have already been reported in the literature [39,43,44]. Deng’s model [43,44] suggested a novel compact model for CNTFETs that is quite similar to the MOSFET model in many aspects. The intrinsic region for the channel is simulated in this model using single-walled CNTFETs as a compact and efficient circuit model. The Deng compact model for CNTFET is relatively very general and can be used for different CNT sizes and types, including both metallic and semiconducting CNTs. Similarly, Deng’s model calculates the CNTFET device parameters by using a substate summation method rather than an integral approach. This substate methodology broadens the device modeling methodologies to include not only CNTFETs but also other 1-D devices such as silicon nanowire FETs. As a result, Deng’s model requires less calculation work, making it more suitable for use with a circuit simulator.

Deng’s model for CNTFET is gaining popularity, as this model incorporates the (i) quasi-ballistic charge transport of CNT channels, (ii) non-ideal behavior by the intrinsic capacitive network, (iii) non-ideal behavior by acoustic scattering of carriers, (iv) non-ideal behavior by optical scattering of phonons in CNTs, (v) non-ideal behavior by the parasitic capacitance between gate/source and gate/drain regions, etc. [12,25,43,44]. Therefore, the proposed Deng model for CNTFET is highly applicable for significant variation in CNT chirality as well as the diameter for CNTFET and can predict the experimental results of CNTFET with more than 90% accuracy [25]. According to the Deng model, the width (*W*) and the energy gap (*E_g_*) for CNTFET can be calculated as [12,45]:

(3) $$W=D_{CNT}+\left(N_{CNT}-1\right)\times S_{CNT}$$

(4) $$E_{g}=\frac{8.84eV}{D_{CNT}}$$

where *N_CNT_* can be identified as the total number of CNTs adjusted between source and drain, while *S_CNT_* is specified as the average pitch between CNTs respectively. Equation (5) expresses the CNTFET transconductance (*g_m_*) [46,47].

(5) $$g_{m}=\left(\frac{1}{1+e^{\frac{2e\varphi_{s}-2aevDS-E_{g}}{2kT}}}-\frac{1}{1+e^{\frac{2e\varphi_{s}-E_{g}}{2kT}}}\right)\frac{1}{R_{q}}\frac{\partial \varphi_{s}}{\partial V_{GS}}$$

where *e* is the electronic charge, *R_q_* is the quantum resistance of CNTFET, $\varphi_{s}$ is the surface potential, -* kT* is the Boltzmann constant, a is the drain optical phonon scattering parameters and *E_g_* is the energy band gap, which is the function of device dimension as seen from Equation (4). Some important CNTFET parameters of Deng’s model for 16 nm technology node are shown in Table 1.

## 3. The Proposed Circuit

**Figure 2:**
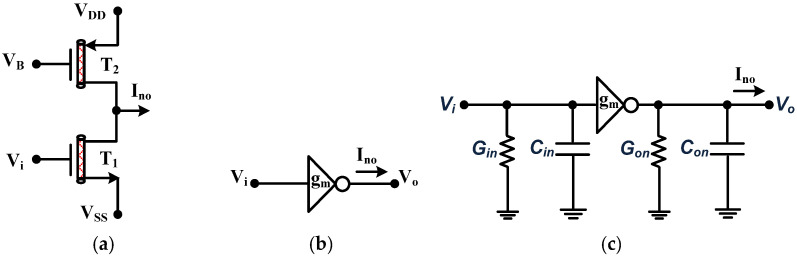
The NT (**a**) transistor realization (**b**) symbol (**c**) equivalent non-ideal model.

**Figure 3:**
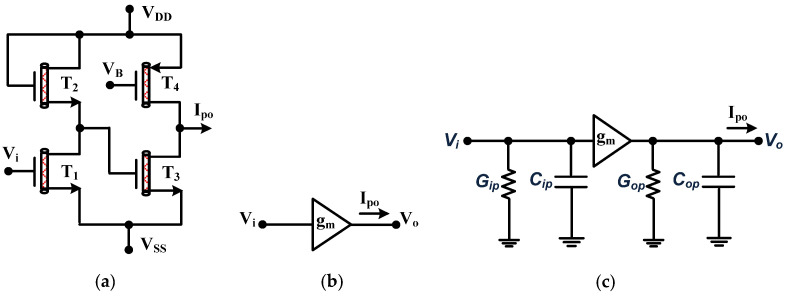
The PT (**a**) transistor realization (**b**) symbol (**c**) equivalent non-ideal model.

**Figure 4:**
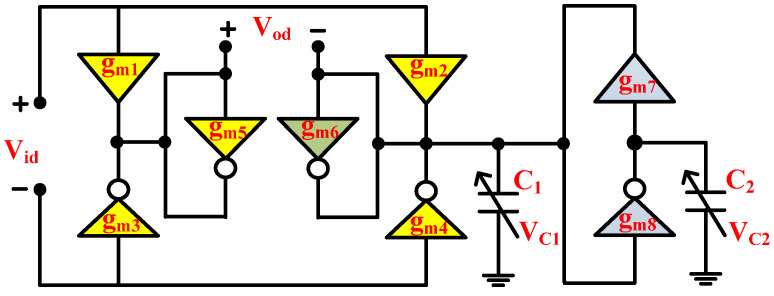
Proposed second-order fully differential APF.

**Figure 5:**
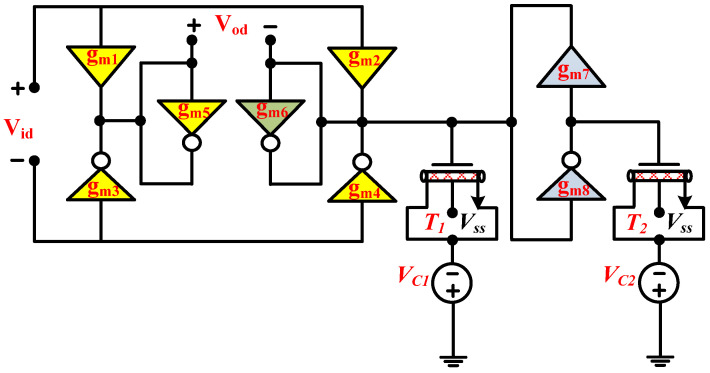
Proposed active-only second-order fully differential APF.

The CNTFET-based negative transconductor (NT) and positive transconductor (PT) are compact active building blocks (ABBs) with single input and output terminals [20]. The NT and PT transistor-based circuit realizations, along with their respective symbols and parasitic models, are given in Figure 2 and Figure 3, respectively [28]. The output current of CNTFET-based NT and PT can be expressed, respectively, as follows:

(6) $$I_{no}=-V_{i}g_{m}$$

(7) $$I_{po}=V_{i}g_{m}$$

where *g_m_* denotes the NT and PT transconductance. Figure 4 shows the proposed second-order fully differential APF, which utilizes three PTs, five NTs and two grounded capacitors. The grounded capacitors make the circuit structure simple and are important with respect to integrated circuit implementation [9]. Moreover, the grounded capacitors can easily absorb the ABBs’ parasitic capacitors. It is important to mention that the utilized ABBs that are employed in the realization of the proposed fully differential APF are based on simple CNTFET circuit configuration. The NT utilizes only two CNTFETs, while the PT utilizes four CNTFETs. Since both ABBs stacked just two transistors between the negative and positive supply rails, these ABBs are suitable for low-voltage operations. Moreover, their constant transconductance over a wide range of frequencies, make them suitable candidates for the design of high-frequency filters [28]. If the utilized capacitors of the proposed APF of Figure 4 were replaced with CNTFET-based varactors [20], the realized APF of Figure 4 reduces to an active-only second-order fully differential APF, as shown in Figure 5.

By ignoring the non-idealities of the utilized transconductors and selecting *g_m_*_1_ = *g_m_*_2_ = *g_m_*_3_ = *g_m_*_4_ = *g_m_*_5_ = *g_mi_* and *g_m_*_7_ = *g_m_*_8_ = *g_mo_*, the routine circuit analysis of the proposed APF of Figure 4 gives the following differential voltage transfer function (VTF):

(8) $$\frac{V_{od}}{V_{id}}=\frac{s^{2}C_{1}C_{2}-sC_{2}\left(g_{mi}-g_{m6}\right)+g_{mo}^{2}}{s^{2}C_{1}C_{2}+sC_{2}g_{m6}+g_{mo}^{2}}$$

If *g_mi_* = 2*g_m_*_6_, a second-order fully differential APF will be realized, with the following VTF:

(9) $$\frac{V_{od}}{V_{id}}=\frac{s^{2}-s\frac{g_{m6}}{C1}+\frac{g_{mo}^{2}}{C_{1}C_{2}}}{s^{2}+s\frac{g_{m6}}{C1}+\frac{g_{mo}^{2}}{C_{1}C_{2}}}$$

From (9), the pole frequency (*ω_p_*) and the zero frequency (*ω_z_*) can be written as:

(10) $$\omega_{p}=\omega_{z}=\omega_{0}=\frac{g_{mo}}{\sqrt{C_{1}C_{2}}}$$

The relative sensitivity (*S*) of pole frequency (*ω*_0_) with respect to transconductance (*g_mo_*) and varactor capacitors (*C*_1_ and *C*_2_) can be found as:

(11) $$S_{g_{mo}}^{\omega_{0}}=1;\;S_{C_{1}}^{\omega_{0}}=S_{C_{2}}^{\omega_{0}}=-\frac{1}{2}$$

Thus, the incremental sensitivities of the proposed filter pole frequency (*ω*_0_) are within unity in magnitude. Also, from (9), the phase angle (ϕ) of the second-order fully differential APF can be derived as:

(12) $$\phi =-2\tan^{-1}\frac{\left(\omega g_{m6}C_{2}\right)}{\left(g_{mo}^{2}-\omega^{2}{C_{1}C}_{2}\right)}$$

From (10) and (12), it can be observed that the proposed filter pole frequency and phase angle are dependent on utilized varactor capacitances (*C*_1_ and *C*_2_). Thus, the tunability can be achieved through the varactor control voltages (*V_C_*_1_ and *V_C_*_2_). The active-only second-order fully differential APF configuration is suitable for integration due to absence of any external passive components.

## 4. Proposed Filter Non-Ideal Analysis

**Figure 6:**
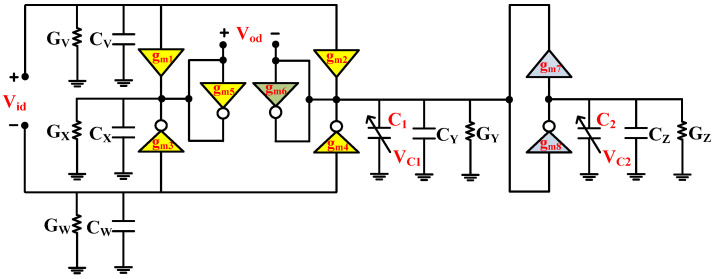
Proposed second-order fully differential APF with parasitics.

The realized filter ideal operation is demonstrated by (8), which does not consider the effect of non-idealities of the utilized PT and NT ABBs. Figure 6 shows the non-ideal equivalent circuit of the proposed second-order fully differential APF of Figure 4, along with the impact of utilized PT and NT non-ideal port parasitics.

In Figure 6:

(13) $$G_{V}=G_{ip1}+G_{ip2}$$

(14) $$C_{V}=C_{ip1}+C_{ip2}$$

(15) $$G_{W}=G_{in4}+G_{in7}$$

(16) $$C_{W}=C_{in4}+C_{in7}$$

(17) $$G_{X}=G_{op1}+G_{on4}+G_{in5}+G_{on5}$$

(18) $$C_{X}=C_{op1}+C_{on4}+C_{in5}+C_{on5}$$

(19) $$G_{Y}=G_{op2}+G_{on7}+G_{in6}+G_{on6}+G_{op3}+G_{in8}$$

(20) $$C_{Y}=C_{op2}+C_{on7}+C_{in6}+C_{on6}+C_{op3}+C_{in8}$$

(21) $$G_{Z}=G_{ip3}+G_{on8}$$

(22) $$C_{Z}=C_{ip3}+C_{on8}$$

By assuming *C*_1_ >> *C_X_*, *C*_1_ >> *C_Y_*, *C*_2_ >> *C_Z_*, *g_m_*_6_ >> *G_X_* and *g_m_*_6_ >> *G_Y_*, the routine analysis of Figure 6 yields the following VTF:

(23) $$\frac{Vod}{Vid}=\frac{s^{2}-s\left(\frac{g_{m6}}{C_{1}}+\frac{G_{Z}}{C_{2}}\right)-\frac{g_{m6}G_{Z}}{C_{1}C_{2}}+\frac{g_{mo}^{2}}{C_{1}C_{2}}}{s^{2}\left(1+\frac{G_{Z}C_{X}}{{2g}_{m6}C_{2}}\right)+s\left(\frac{g_{m6}}{C_{1}}+\frac{G_{z}}{C_{2}}+\frac{g_{mo}^{2}C_{X}}{{2g}_{m6}C_{1}C_{2}}\right)+\frac{g_{m6}G_{Z}}{C_{1}C_{2}}+\frac{g_{mo}^{2}}{C_{1}C_{2}}}$$

The zero and pole frequencies are slightly affected due to non-idealities. However, it is evident from [28] that the parasitic capacitance (*C_X_*) is on the order of aF and parasitic conductance (*G_Z_*) is on the order of nS. Since *C_X_* and *G_Z_* are small enough, by neglecting these parasitics, (23) will be reduced to (9). Thus, the parasitics- role is almost insignificant.

## 5. Design and Verifications

**Figure 7:**
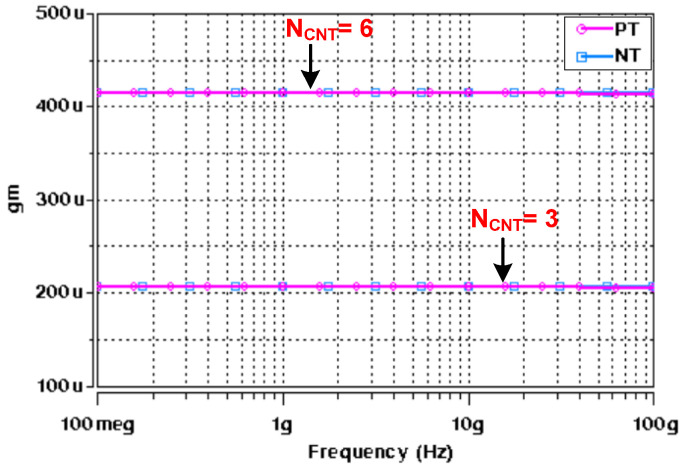
AC response of transconductance gain (*g_m_*) with different *N_CNT_*.

**Figure 8:**
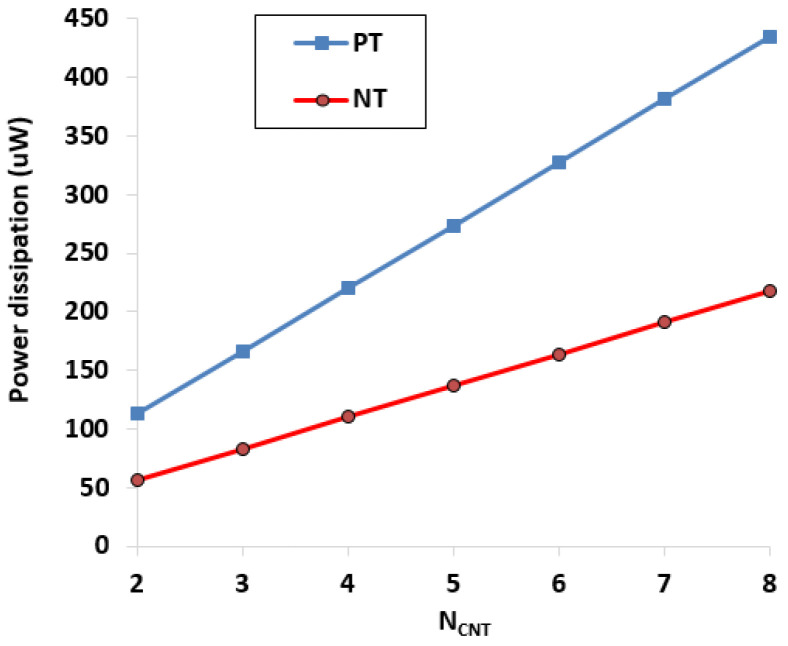
N_CNTs_ impact on power dissipation of NT and PT.

To justify the proposed theory, the realized filters of Figure 4 and Figure 5 were designed and verified through HSPICE-based simulations. Deng’s CNTFET model was utilized with supply voltage ±0.7 V. The Deng CNTFET model parameters of Table 1 are used for simulations. The simulations were conducted for different number of CNTs (*N_CNT_*). Figure 7 shows the AC response of the transconductance of the CNTFET-based PT and NT ABBs for *N_CNT_* = 3 and *N_CNT_* = 6 only. It was observed that with increasing *N_CNT_*, the transconductance of the respective ABB increases due to increase in the width of transistor [28]. Moreover, the constant magnitude of the transconductance over a wide frequency band makes these ABBs suitable for a higher-frequency range of applications [20]. The power dissipation also increases with the utilization of more *N_CNTs_* in the NT and PT ABBs. Figure 8 demonstrates the impact of *N_CNTs_* on power dissipation of these ABBs.

**Figure 9:**
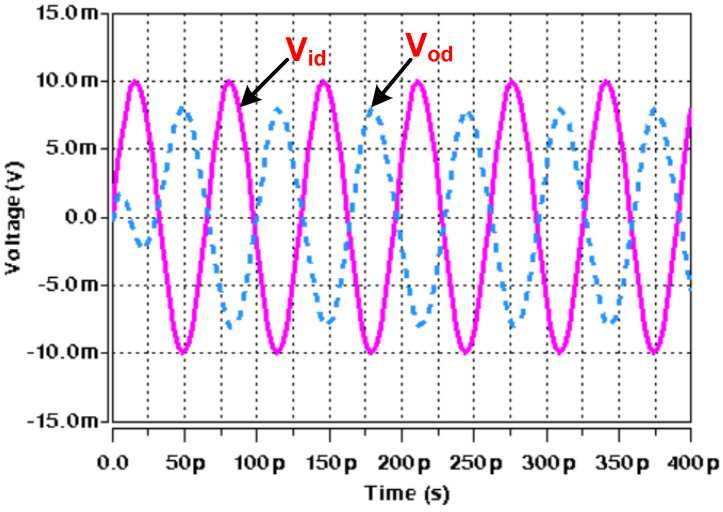
Transient response of second-order fully differential APF at *f*_0_ = 15.0 GHz.

**Figure 10:**
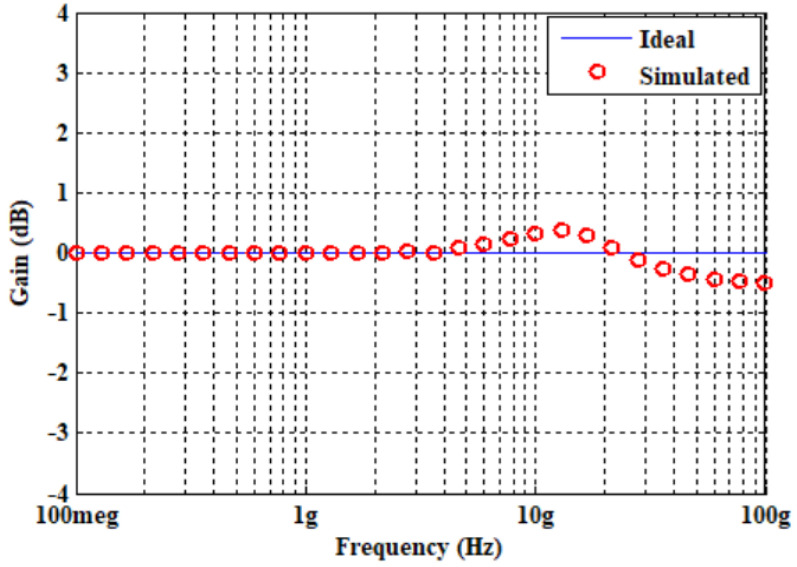
Ideal versus simulated-frequency response of voltage gain of second-order fully differential APF.

**Figure 11:**
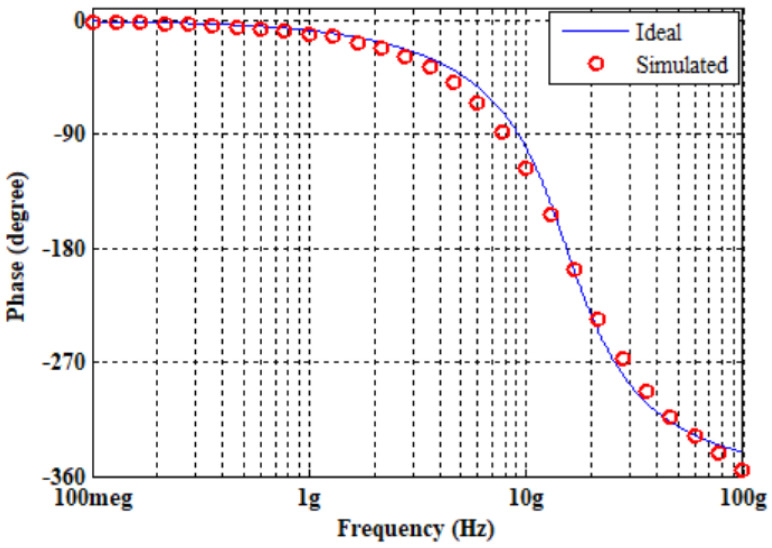
Ideal versus simulated-frequency response of phase gain of second-order fully differential APF.

**Figure 12:**
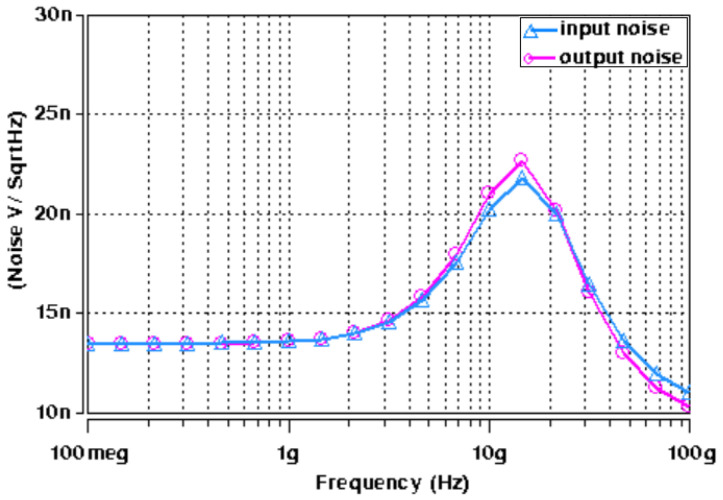
Input and output noise of second-order fully differential APF.

Initially, the proposed APF of Figure 4 was designed and simulated for a pole frequency of 15 GHz. The transconductance of the employed ABBs was set to fulfill the primary requirement (*g_mi_* = 2*g_m_*_6_), as demonstrated by (8) and (9). Table 2 shows the description of the transconductor designed values. For the desired pole frequency of *f*_0_ = 15.0 GHz, with capacitor *C*_1_ = 2 fF, (10) yields *C*_2_ = 1 fF. HSPICE simulation of the designed second-order fully differential APF results in the transient response as shown in Figure 9. The input and output differential voltage shows a phase shift of 180° at the designed *f*_0_ = 15.0 GHz, which demonstrates the operation of circuit as a second-order APF. The theoretical and simulated frequency responses of the gain and phase are given in Figure 10 and Figure 11, respectively, which show that the second-order fully differential APF simulation outcomes are almost in line with the theoretical prediction. The power dissipation of the proposed second-order fully differential APF was observed as 1.40 mW. Figure 12 demonstrates the input noise and output noise simulation results, which are obtained as 21.69 $nV\sqrt{Hz}$ and 22.54 $nV\sqrt{Hz}$, respectively, at the designed *f*_0_ = 15.0 GHz, which are significantly low in magnitude.

**Figure 13:**
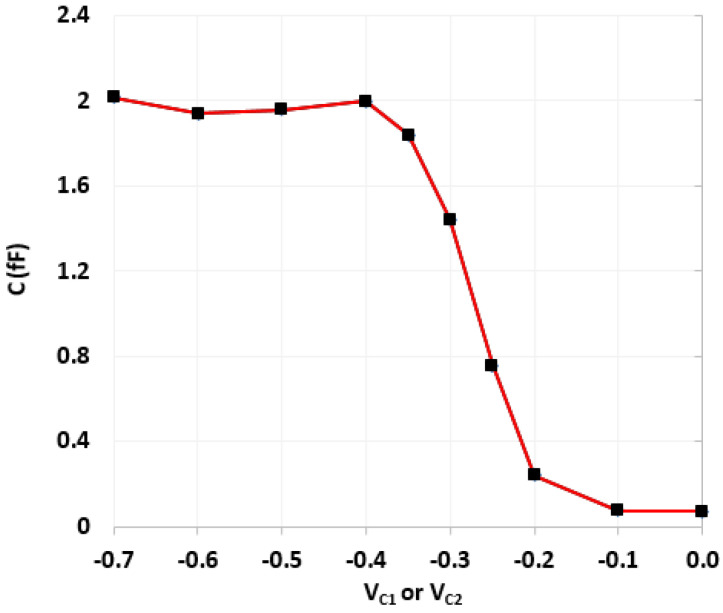
Varactor capacitance variations with control voltages V_c1_ and V_c2_.

**Figure 14:**
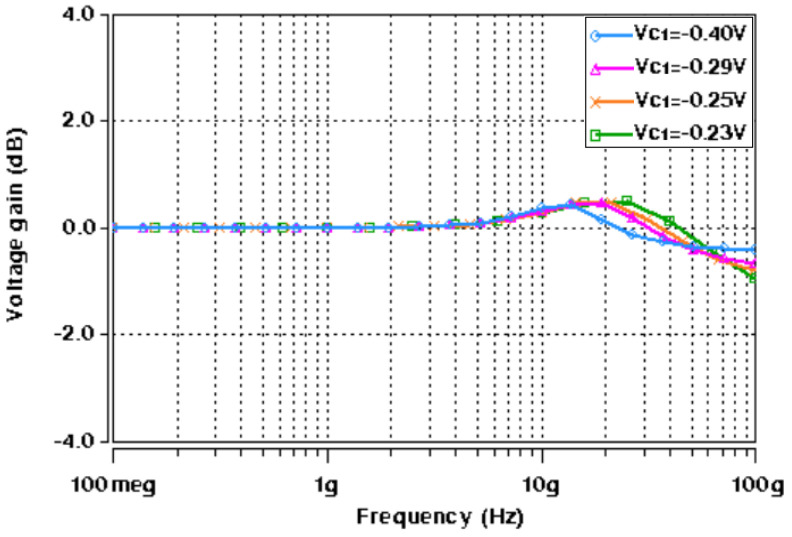
AC response of proposed active-only APF at different values of *V_C_*_1_ (at *V_C_*_2_ = −0.27 V).

**Figure 15:**
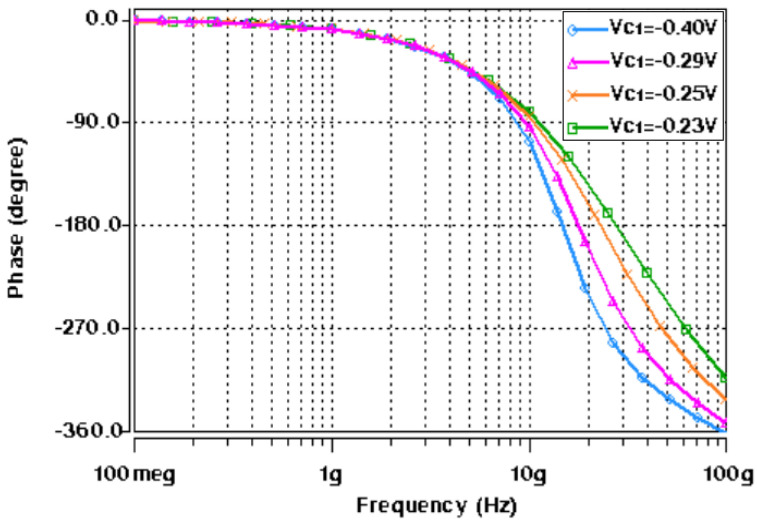
AC response of proposed active-only APF phase at different values of *V_C_*_1_ (at *V_C_*_2_ = −0.27 V).

**Figure 16:**
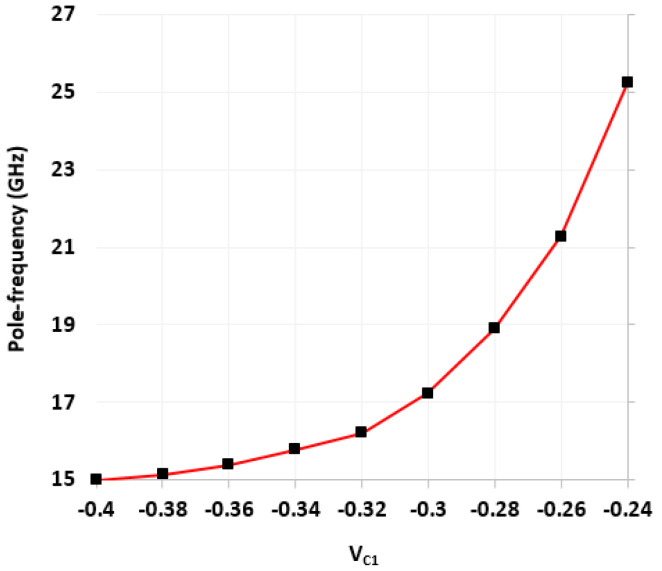
Impact of variation of varactor voltage V_c1_ on pole frequency of fully differential APF (at *V_C_*_2_ = −0.27 V).

Next, the realized active-only second-order fully differential APF of Figure 5 was also simulated with the CNTFET parameters of Table 1 and Table 2. Figure 13 shows the impact of variation of control voltages (*V_C_*_1_ and *V_C_*_2_) on the utilized varactor capacitance. The CNTFET parameters of Table 1 are utilized for both varactors, along with *N_CNT_* = 229. These varactor capacitance variations are used for tunability of the proposed fully differential APF. Initially the varactor voltages *V_C_*_1_ and *V_C_*_2_ are set to −0.4 V and −0.27 V, respectively, which results in *C*_1_ = 2 fF and *C*_2_ = 1 fF. For simplicity, the varactor voltage *V_C_*_2_ was kept constant at −0.27 V, while the varactor voltage *V_C_*_1_ was varied from −0.4 V to −0.23 V to demonstrate the tunability. Figure 14 demonstrates the frequency response of transfer gain of the active-only second-order fully differential APF for selected values of varactor voltage *V_C_*_1_. Figure 15 shows the frequency response of the phase angle for selected values of varactor voltage *V_C_*_1_. It was observed from Figure 15 that by tuning the *V_C_*_1_ from −0.4 V to −0.23 V, the active-only second-order fully differential APF pole frequency changes from 15 GHz to 27.5 GHz, which maintains the same phase angle of 180°. The impact of varactor voltage *V_C_*_1_ on the proposed filter pole frequency is demonstrated in Figure 16. It is noticed that by decreasing the *V_C_*_1_ below −0.23 V, the overall parasitics of the utilized ABBs degrades the gain and phase response of the active-only fully differential APF.

## 6. Comparison of the Proposed Filters with Other FDSOAPFs

Several fully differential APF are available in the open literature [2,13,14,15,16,17,18,19,20,21,22,23,24]. However, the majority of these circuit configurations are for first-order APFs [13,14,15,16,17,18,19,20,21,22]. Very few second-order fully differential APFs are available in the open literature [2,23,24]. It is also to be noted that CNTFET-based second-order fully differential APFs are not observed by the authors in the available technical literature. Table 3 shows a comparison of the realized filters with other relevant reported second-order fully differential APFs. It can be seen that the proposed filter of Figure 4 uses a minimum number of passive components compared with the reported APFs [2,23,24]. Moreover, the proposed second-order fully differential APF uses grounded capacitors like [24], whereas the APFs [2,23] are based on floating passive components. The reported APFs [2,23,24] have frequency limitations, where the frequency range is limited to a few KHz or MHz, whereas the proposed APF operates in the GHz range. The active-only second-order fully differential APF of Figure 5, which is obtained from the realized APF of Figure 4, does not utilize any passive components and is thus suitable for integration. It is also to be noted that the APFs of [2,23,24] are not tunable; however, the realized active-only second-order fully differential APF is tunable for a wide frequency range. Some prominent features of the proposed active-only APF circuit include electronic tunability, low operating supply voltages, low power consumption, and wider operational frequency ranges. Moreover, the active-only APF circuit demonstrates exceptional transient AC characteristics and works equally well at deep-submicron technology nodes.

## 7. Conclusions

In this paper, new CNTFET-based second-order fully differential APFs are proposed. The realized circuits operate in voltage mode and use compact CNTFET-based positive and negative transconductors. The first circuit uses three PTs, five NTs and two grounded capacitors. The active-only second-order fully differential APF is obtained from the first APF circuit topology. The active-only APF tunability is achieved by utilizing voltage-controlled varactors. Both fully differential filter topologies are simulated by utilizing Deng’s well known CNTFET model in HSPICE. The proposed topologies show excellent transient, gain and phase characteristics and work equally well in the GHz frequency range. The active-only fully differential filter topology shows a wide tunable pole frequency. In addition, the proposed APFs work on low supply voltages and consume low power. Thus, both the realized second-order fully differential APF circuits are attractive candidates for low-voltage and low-power applications in the GHz frequency range. The major advantage of the proposed fully differential-second order APF is its operation in the GHz range and tunability over a wide range of frequencies. This advantage has a strong potential for CNTFET-based realization of active filters in the nanometer regime. Among many, a potential application of such nanometer regimes has emerged in the evolving 5th Generation (5G) of land mobile radio frequency cellular communication systems for addressing the increasing capacity demands. It will be interesting to experimentally validate the proposed fully differential APF; however, due to unavailability of the required resources, experimental verification has not been possible. The fully differential APF physical realization may be a vital direction for future extension of the proposed work. The work in this paper is also helpful for the subsequent research of radio frequency beamforming and the realization of oscillator circuits. The HSPICE simulation results on the realized second-order fully differential APF circuits support the theoretical predications.

## Figures and Tables

**Table 1:** Deng’s CNTFET model parameters.

CNTFET-Parameters	Parameter Description	Value
*V*	Power supply	±0.7 V
*L_g_*	Channel length	16 nm
*L_ceff_*	Mean free path	200 nm
*K_ox_*	Dielectric constant	25
*L_s_*/*L_d_*	Source/drain side length of doped CNT	16 nm
*T_ox_*	Oxide thickness	3 nm
*K_sub_*	Bottom gate dielectric constant	SiO_2_ (4)
*S_CNT_*	Inter-CNT Pitch	10 nm
*D_CNT_*	CNT-Diameter	1.5 nm
*N_CNT_*	Total CNTs utilized per CNTFET	-

- variable parameter.

**Table 2:** Designed transconductance values with respect to N_CNTs_.

Transconductance	Value (μS)	*N_CNTs_*
*g_m_*_1_	414.1	6
*g_m_*_2_	414.1	6
*g_m_*_3_	414.1	6
*g_m_*_4_	414.1	6
*g_m_*_5_	414.1	6
*g_m_*_6_	207.1	3
*g_m_*_7_	136.6	2
*g_m_*_8_	136.6	2

**Table 3:** Comparison with other available active-only second-order fully differential APFs.

Reference	[2]	[23]	[24]	This Work Figure 4	This Work Figure 5
Technology	CMOS	CMOS	CMOS	CNTFET	CNTFET
node	0.35 μm	0.5 μm	0.35 μm	16 nm	16 nm
Voltage (V)	2.5 V	2.5	1.65	0.7	0.7
ABB utilized	CCII-	DVCC	(DDCC & CCII)	(PT & NT)	(PT & NT)
No. of R/C	3/3	3/3	3/2	0/2	0/0
Tunability	No	No	No	No	Yes
Pole frequency (Hz)	195 K	3.18 M	100 K	15.0 G	15.0 G to 27.5 G
Power (mW)	-	-	-	1.40	1.59
(Grounded passive components)	No	No	Yes	Yes	~

- Not available, ~ Not applicable.

## Data Availability

The data that support the findings of this study are available from the corresponding author upon reasonable request.
